# Hybrid nanofluid flow within cooling tube of photovoltaic-thermoelectric solar unit

**DOI:** 10.1038/s41598-023-35428-6

**Published:** 2023-05-21

**Authors:** Z. Khalili, M. Sheikholeslami, Ladan Momayez

**Affiliations:** 1 Department of Mechanical Engineering, Babol Noshirvani University of Technology, Babol, Islamic Republic of Iran; 2 Renewable Energy Systems and Nanofluid Applications in Heat Transfer Laboratory, Babol Noshirvani University of Technology, Babol, Islamic Republic of Iran; 3 Department of Engineering and Computer Science, University of Pittsburgh at Johnstown, Johnstown, PA USA

**Keywords:** Nanoscience and technology, Nanoscale materials

## Abstract

In this work, the thermoelectric generator (TEG) layer has been combined with conventional layers of photovoltaic-thermal (PVT) modules to use the waste heat and increase the efficiency. To reduce the cell temperature, there exists a cooling duct in the bottom of the PVT-TEG unit. Type of fluid within the duct and structure of duct can change the performance of the system. So, hybrid nanofluid (mixture of Fe_3_O_4_ and MWCNT with water) has been replaced instead of pure water and three various configurations of cross section [STR1 (circular), STR2 (rhombus), STR3 (elliptic)] have been implemented. Through the tube incompressible laminar flow of hybrid nanofluid has been solved while in solid layers of panel, pure conduction equation has been simulated involving heat sources resulting from optical analysis. According to simulations, the third structure (elliptic) has the best performance and rise of inlet velocity causes overall performance to enhance about 6.29%. The values of thermal and electrical performances for elliptic design with equal fractions of nanoparticles are 14.56% and 55.42%, respectively. With the best design, electrical efficiency improves about 16.2% in comparison with an uncooled system.

## Introduction

Energy holds significant economic importance for any country, as it is not only crucial for industries, but also for meeting the domestic needs of society. This energy can take various forms, such as electricity, chemicals, heat, and others. Traditionally, fossil fuels have been used to fulfill these energy demands, but they are finite resources that cannot be easily replenished. The rate at which humans consume fossil fuels far exceeds the rate at which they are naturally substituted^1^. Therefore, finding sustainable alternatives to fossil fuels is essential for meeting our long-term energy needs. Sustainable energy is a pivotal issue that has the potential to bring about positive change in the present situation^2^. Fossil fuels not only contribute to environmental pollution, but also face the challenge of depletion. So, to decrease the environmental impact of such sources, the request for renewable energy is increasing to meet the growing energy needs. As the cost of solar energy drops below that of fossil fuels, the demand for fossil fuels tends to decrease. Solar energy can be harnessed through various systems, including Photovoltaic Thermal (PVT) units for producing both heat and electricity from solar energy^3^. PV units are applied to convert incident radiation into electricity and only 20% of the whole energy of sunlight can be converted and remaining is wasted^4^. However, elevated operating temperatures can lead to a reduction in the conversion rate and this temperature increase can result in damage to the structural integrity of the solar panels^5^. Efforts to augment the electrical performance (*η*_*el*_) of PV panels involve reducing their operating temperature, which can be reached through the employ of a thermal absorber unit. Researchers have explored a method called PVT unit, to lower cell temperature^6^. The PVT system enables simultaneous generation of electricity and heat^7,8^. Elqady et al.^9^ investigated a research to optimize the dimensions of a heat sink for improving cooling performance of solar panels. Their findings identified a duct with optimal design points then they employed in a 3D model to assess the effectiveness of a PVT. The greatest electricity performance achieved was 17.45%, which demonstrated a significant improvement of nearly 40% compared to a typical CPV/T system. Raza et al.^10^ have presented a computational methodology for designing a high-performance composite material to be used as the backside of a concentrated PV (CPV) unit. The proposed composite shows promising potential and results in a 4.3% enhancement in electrical output and improved module durability. Li et al.^11^ presented a novel and versatile approach for cooling photovoltaic panels. They found that performance of PV enhances about 19% with employing the proposed system.

Through ongoing research on fluid properties, water can be modified to enhance its heat removal capabilities for photovoltaic (PV) cells. This can be achieved by incorporating nanoparticles into water to increase its thermal conductivity^12^. Nanofluid, which is a type of heat transfer medium composed of nanometer-sized engineering materials mixed with a base fluid, has garnered significant attention from researchers due to its performance in various usages^13^. Nanofluids have gained attention as promising cooling techniques for PVT. Researchers have experimented with different nanofluids in different structures of PVT systems to optimize their efficiency and establish an effective system with improved overall performance^14^. A research managed by Bassam et al.^15^ examined the efficiency of a hybrid PVT in existence of micro fins and turbulator. The reported *η*_*el*_ of the unit was 10.8% and the maximum thermal performance of the unit was 83.3%. The optimal operating conditions for a PVT system with CuO nanofluid were scrutinized by Madas et al.^16^. The outputs showed that increasing the nanofluid fraction resulted in a 1.11% and 3.3% increase in electrical and thermal performances. Abadeh et al.^17^ studied the economic analysis of solar system in the existence of various types of nanofluid as coolants. Their findings revealed that the addition of nanofluids significantly improved the payback period. Moreover, from an environmental perspective, outputs demonstrated that the proposed unit decreased emission production about 17% compared to a PV unit. Nasrin et al.^18^ tested an indoor experiment on a PV and they applied MWCNT-water as testing fluid. They reported the overall efficiency can reach 87.65%. Khan et al.^19^ evaluated the beahvior of PVT system utilizing a serpentine pipe. Their study revealed that PVT systems utilizing hybrid nanofluids exhibited 10.5% higher thermal performance compared to Iron oxide–water. Alktranee et al.^20^ employed a research to scrutinize the impact of using nanofluid on efficiency of PVT system. They utilized tungsten trioxide and showed that cell temperature reduces about 21.4%. Tembhare et al.^21^ reported a review of nanomaterial and their properties for solar thermal applications. They analyzed various studies on solar thermal systems that use nanofluids. The researchers found that nanofluids, due to their superior heat transfer properties, offer significant potential for solar applications. Nanofluids, with dispersed nanoparticles that exhibit high thermal conductivity, have the capability to transport heat efficiently. Du et al.^22^ incorporated a filter containing plasmonic nanofluids into their PVT system to make use of the entire solar spectrum. Additionally, they employed aerogel glazing and observed a 13.3% increase in exergy performance compared to the previous system.

To enhance the performance of solar energy utilization, there are significant researches on PVT, which are efficient and cost-effective technologies. Furthermore, there has been a growing interest in hybrid systems combining photovoltaic with thermoelectric generators (PV-TEG)^23^. Attempts have been made to combine PV and TE technologies. Despite the benefits of thermoelectric generators (TEG) in converting waste heat into electricity. TE modules offer several advantages, such as being environmentally friendly, simple, silent, and durable. However, their efficiency is relatively low. While PV cells cover the visible and ultraviolet ranges of the solar irradiation, TEG modules can utilize the infrared part, resulting in a more comprehensive energy harvesting from whole sunlight^24^. By utilizing the Seebeck effect, a thermoelectric generator (TEG) module is able to generate electrical power by harnessing temperature differences. In a PV/TEG hybrid unit, the PV-temperature increases as solar radiation intensifies. Subsequently, the TEG converts the temperature gradient into electrical energy, following the principles of the Seebeck impact^25–27^. In a simulation conducted by Rejeb et al.^28^, a comparison was made between a CPVT unit and a CPVT/TE unit. They proved that the CPVT/TE system with nanofluid generated 11.15% more overall electrical power in summer compared to the CPVT unit. Chen et al.^29^ scrutinized a combination of TEG, PV and solar selective absorber (SSA). Their output exhibited a 9.89% increase in energy efficiency. Lekbir et al.^30^ scrutinized a CPVT-TE unit that utilized a nanofluid cooling channel. The outcomes revealed that *η*_*el*_ of this unit was 8.4% greater compared to CPVT-TE with water cooling. Shittu et al.^31^ scrutinized a simulation research on a PVT-TEG unit in existence of heat pipe (HP). The outcomes revealed that the suggested system's performance was 1.47 times greater than that of the system without HP. A prototype of a CPVT/TE unit was evaluated by Indira et al.^32^ under outdoor conditions. They found that the highest electrical performance of 4.86% was achieved.

According to previous study, changing the layers of PV modules and utilizing cooling systems can change the efficiency. Some researchers suggested using TEG to employ the waste heat resulting from wavelengths of sunlight which cannot convert to electricity via silicon layer. In current paper, a PV module was joined with TEG layer and cooling duct with various configurations have been applied for managing the cell temperature. The hybrid nanoparticles (Fe_3_O_4_—MWCNT) were dispersed within water. The influences of fraction ratio of components of hybrid nanofluid as well as inlet velocity of testing fluid have been examined via numerical modeling. Three geometries for cooling ducts have been incorporated to find the best design. Also, the influence of the amount of solar irradiation has been analyzed. The governing equations and utilized assumptions have been summarized in section "The description of PVT-TEG system and governing equations". The outputs of simulations have been classified in section "Results and discussion" to find the case with best performance. Conclusion section has been presented as the last part of this article.

## The description of PVT-TEG system and governing equations

**Figure 1:**
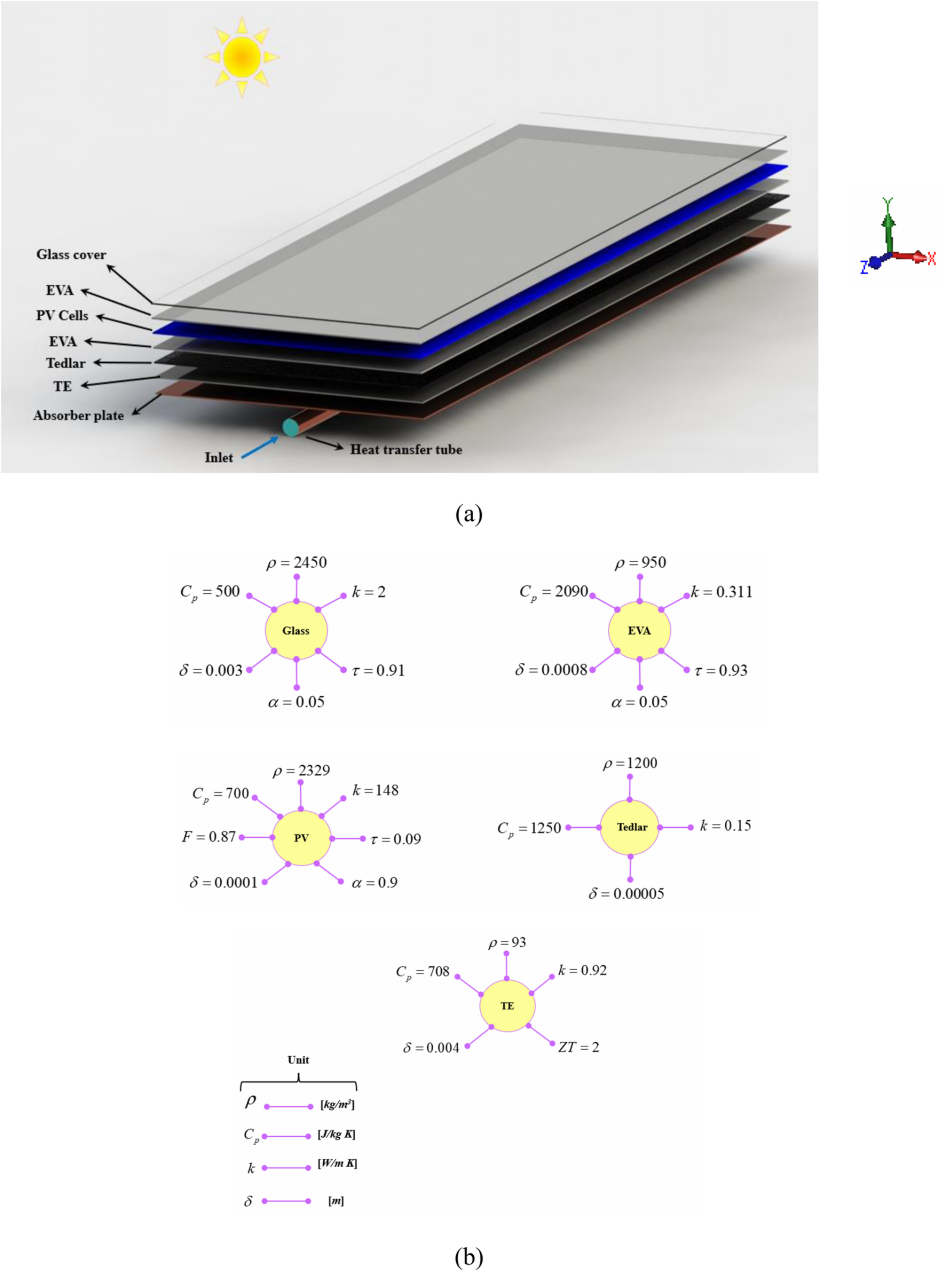
(**a**) PVT combined with TEG, and (**b**) geometrical and thermophysical specifications of the solid regions.

**Figure 2:**
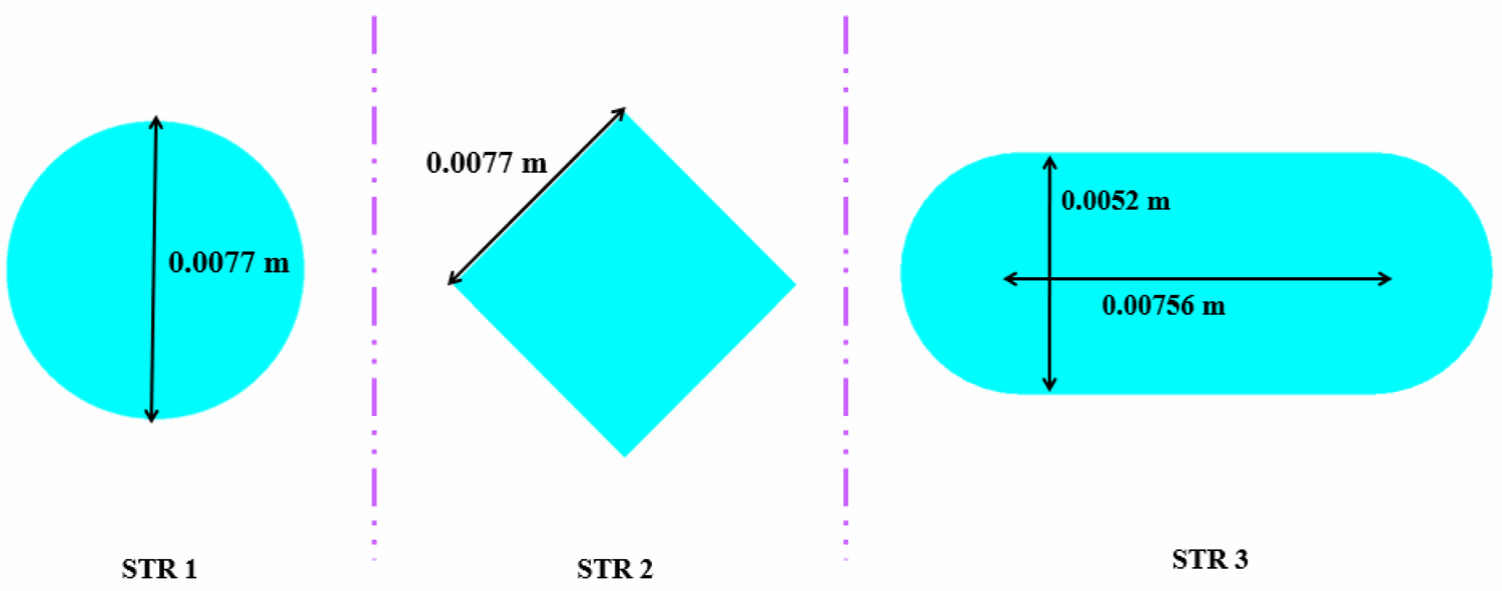
The proposed structures for the cross section of cooling duct.

The polycrystalline silicon panel with 72 cells and critical temperature of 85 °C has been selected in this study and the associated data for dimensions and properties of layers are the same of Ref.^18^. Different layers have been shown in Fig. 1 and it can be seen that TEG layer exists above absorber layer. Thicknesses of each layer and their properties have been mentioned in the first figure. Adding a TEG layer makes the output of the system increase. For preparing the cooling equipment, the cooling duct has been located in the bottom of the absorber. As mentioned in Fig. 2, three configurations (STR1 (circular), STR2 (rhombus), and STR3 (elliptic)) have been implemented.

**Figure 3:**
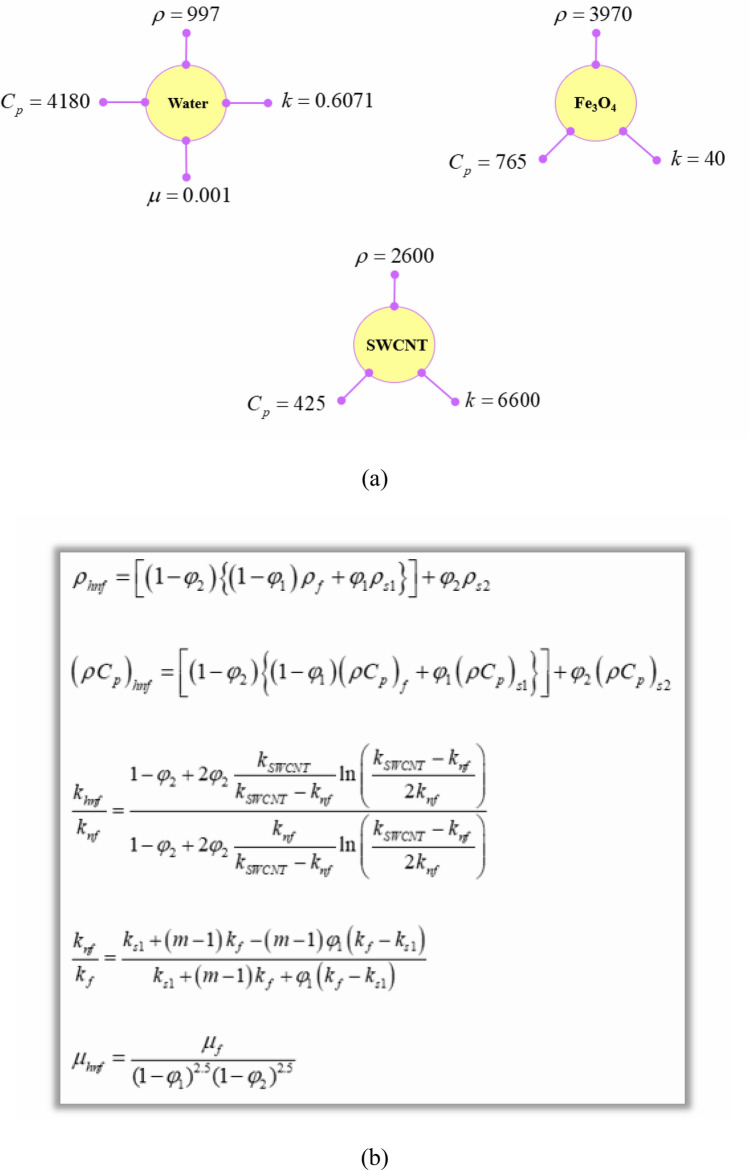
(**a**) Thermophysical specifications of the H_2_O and nano-powders, and (**b**) the governing equations for thermophysical properties of Fe_3_O_4_-SWCNT/water hybrid nanofluid.

Hybrid nanofluids are becoming increasingly popular due to their potential to augment the useful heat. Unlike mono-nanofluids, hybrid nanofluids can lead to better thermal conductivity, viscosity, and stability. Moreover, hybrid nanofluids offer greater design flexibility and can be customized to meet specific application needs. Therefore, utilizing hybrid nanofluids has been utilized in current work (see Fig. 3). The hybrid nanoparticles are a mixture of Fe_3_O_4_-SWCNT and water has been applied as base fluid. The properties of components and formulas for calculating the hybrid nanofluid features have been shown in Fig. 3 and for more explanation exist in Ref.^33^.

Three dimensional simulations have been applied and symmetric conditions have been utilized and one duct has been simulated to reduce the computing price. The flow of hybrid nanofluid must be simulated based on below equations^34^:

1 $$\nabla \cdot \vec{V}_{hnf} = 0$$

2 $$\vec{V}_{hnf} \cdot \nabla \left( {\rho_{hnf} \vec{V}_{hnf} } \right) = - \nabla P + \mu_{hnf} \nabla^{2} \vec{V}_{hnf}$$

3 $$\vec{V}_{hnf} \cdot \nabla (\rho_{hnf} T_{hnf} ) = \nabla \cdot \left( {\frac{{k_{hnf} }}{{C_{p,hnf} }}\nabla T_{hnf} } \right)$$

For simulating the various layers which have been shown in Fig. 1, below equation should be solved^34^:

4 $$\nabla \cdot (k_{R} \nabla T_{R} ) + \dot{\Re }_{R} = 0$$

The index (R) denotes the name of layers. The second term can be calculated as below^35,36^:

5 $$\dot{\Re }_{Glass} = GA\alpha_{g}$$

6 $$\dot{\Re }_{EVA1} = GA\tau_{g} \alpha_{EVA}$$

7 $$\dot{\Re }_{silicon} = GA\tau_{g} \tau_{EVA} F\alpha_{silicon} (1 - \eta_{PV} )$$

8 $$\dot{\Re }_{EVA2} = GA\tau_{g} \tau_{EVA} [(1 - F) + F\tau_{silicon} ]\alpha_{EVA}$$

9 $$\dot{\Re }_{Tedlar} = \dot{\Re }_{TEG} = \dot{\Re }_{Absrober} = 0$$

Thermal performance of unit can be achieved according to below formula^37^:

10 $$\eta_{th} = \left[ {\dot{m}_{hnf} C_{p,hnf} (T_{out} - T_{in} )} \right]/\left( {GA\tau_{g} \tau_{EVA} F\alpha_{silicon} } \right)$$

To calculate PV electrical performance (*η*_*PV*_), the following equation must be used^37^:

11 $$\eta_{PV} = \eta_{ref} [1 - \beta_{ref} (T_{PV} - T_{ref} )]$$

To calculate the energy consumption of pump, below equation can be used^38^:

12 $$E_{pump} = \frac{{\Delta P_{hnf} }}{{\eta_{pump} \rho_{hnf} }}\dot{m}_{hnf} ,\quad \eta_{pump} = 0.8$$

TEG layer can convert part of waste heat to electricity; the efficiency of TEG can be calculated as^39^:

13 $$\begin{aligned} \eta_{TEG} & = \frac{{Q_{TEG} }}{{\left( {GA\tau_{g} \tau_{EVA} F\alpha_{silicon} } \right)}}\left( {\frac{{T_{H} - T_{L} }}{{T_{H} }}\frac{{\sqrt {ZT + 1} - 1}}{{T_{L} /T_{H} + \sqrt {ZT + 1} }}} \right), \\ ZT & = \left( {0.5\sigma \left( {T_{H} + T_{C} } \right)\frac{{S^{2} }}{k}} \right) \\ \end{aligned}$$

To evaluate the system in view overall electrical efficiency, below equation can be applied^38,39^:

14 $$\eta_{el} = \eta_{PV} - \frac{{E_{Pump} }}{{\left( {GA\tau_{g} \tau_{EVA} F\alpha_{silicon} } \right)}} + \eta_{TEG}$$

In this numerical study, ANSYS FLUENT 18.2 was utilized to simulate the PVT-TE system. The chosen method for pressure–velocity coupling was the SIMPLE method. The gradient spatial discretization was achieved using the Least Squares Cell-based method. The 2nd order method was selected for solving the pressure equation. The residual amounts of the continuity touched 10^−5^, and the residual amounts of the energy equation reached 10^−6^.

## Results and discussion

The combination of a PVT unit, TEG layer, and hybrid nanofluid cooling in the bottom cooling duct offers various benefits. Firstly, such unit can provide both heat and electricity while converting waste heat into additional electricity through TEG, resulting in increased efficiency and energy output. Secondly, incorporating hybrid nanofluid cooling in the bottom cooling duct improves thermal management, dissipates heat effectively, and reduces thermal stress on the system. This results in enhanced system reliability and lifespan. Additionally, nanofluid cooling improves heat transfer coefficients, and provides further efficiency gains. In summary, combining PVT, TEG, and nanofluid cooling significantly improves energy efficiency, thermal management, and system reliability. The working fluid consists of H_2_O and combination of Fe_3_O_4_ and MWCNT as hybrid nano-powders. The geometry of the duct at the bottom of a PV system is essential for effective cooling and heat dissipation. Properly designed duct geometry can enhance the flow of the cooling fluid and improve heat transfer, resulting in lower operating temperatures and better system performance. The shape and size of the duct can also affect the pressure drop and flow rate, which are critical factors for maintaining the system's optimal performance. Additionally, the duct's shape can influence the distribution of the fluid, which ultimately determines the cooling performance of the system. Therefore, proper consideration of the geometry of the duct at the bottom of a PV system is crucial for ensuring efficient operation and maximizing the system's lifespan. According to this fact, three various geometries for cooling duct have been suggested in present work with considering the same hydraulic diameter (STR1 (circular), STR2 (rhombus), STR3 (elliptic)). The influences of various fractions of components of hybrid nanofluid and inlet velocity (V_in_ = 0.065 to 0.17 m/s) have been scrutinized.

### Selection of best grid

**Figure 4:**
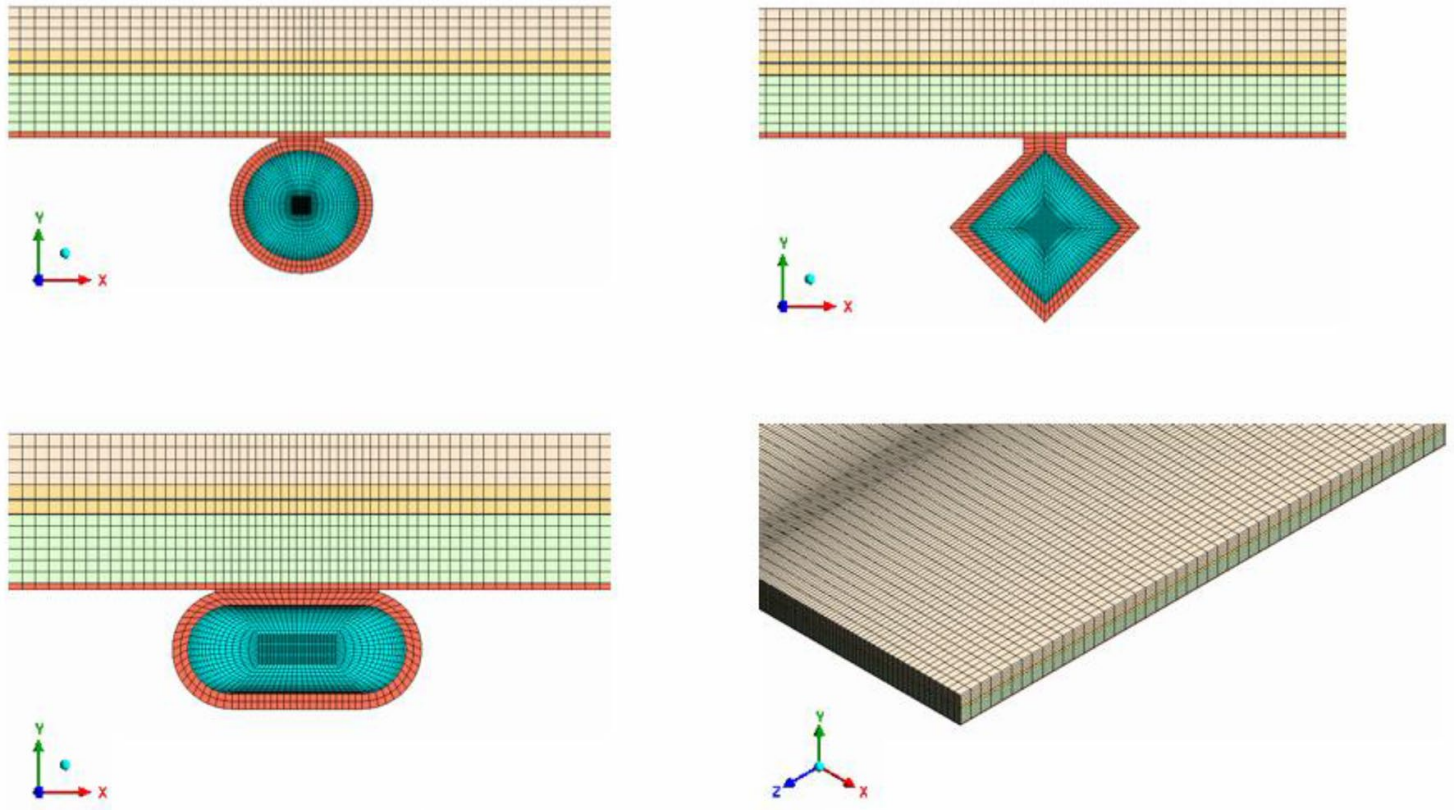
The structured mesh for the current system.

**Figure 5:**
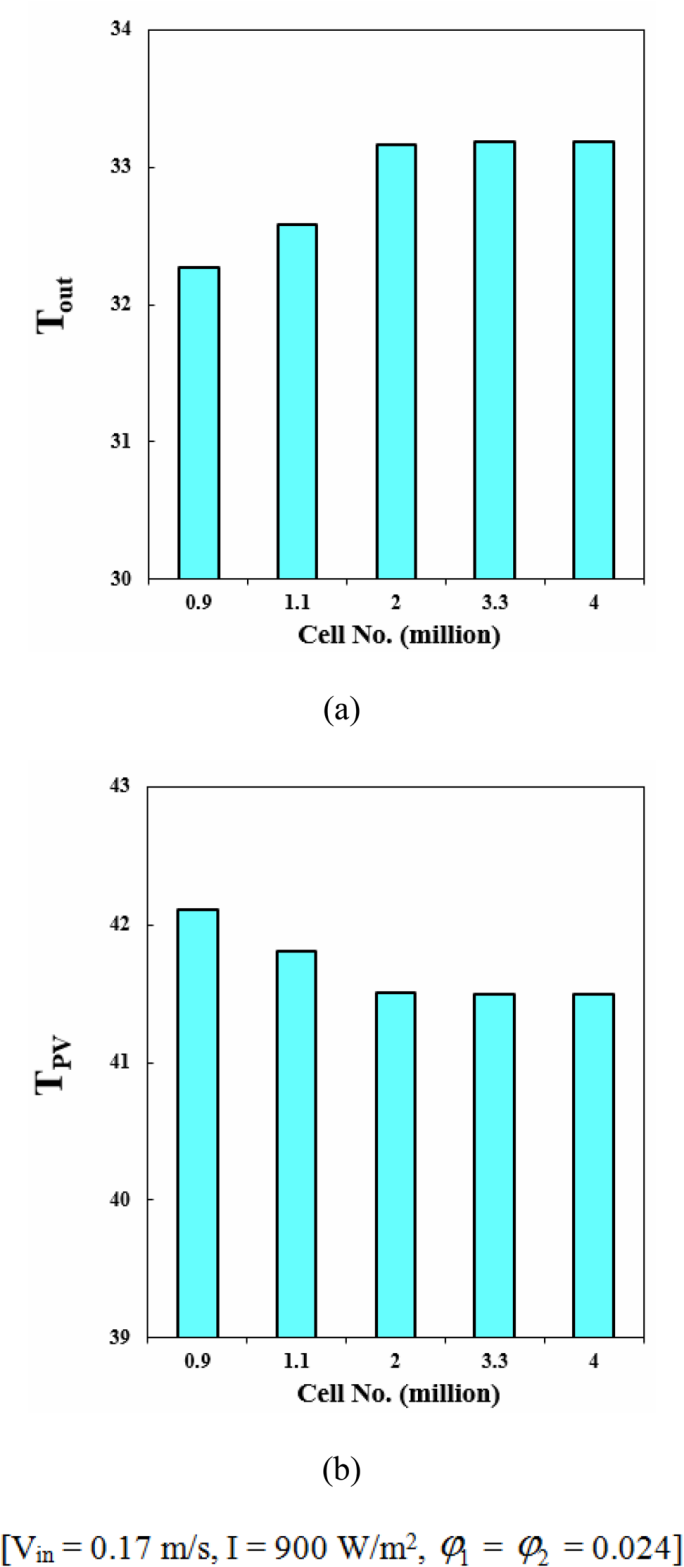
Grid independence study for STR1 reporting (**a**) T_out_, (**b**) T_PV_.

Achieving mesh independence is a critical step in numerical simulations as it ensures precise and trustworthy results. The process involves modifying the mesh density to determine the minimum resolution necessary for accuracy. The accuracy of the simulation output is heavily influenced by the mesh density, and using a mesh that is too coarse or fine may lead to unreliable results. Thus, obtaining mesh independence is crucial in producing accurate simulations, which helps engineers make informed design decisions, enhance system performance, and ensure reliability. A structured grid is a mesh system where the cells are arranged in a regular pattern and can be identified by their indices. It has advantages such as better accuracy and stability in numerical simulations, faster convergence rates, and ease of implementation for structured geometries. So, structured mesh has been applied in present modeling as illustrated in Fig. 4. When selecting the optimal number of grid cells, two criteria were taken into consideration: T_out_ and T_PV_. Five different grid resolutions were evaluated, and the grid with 3.3 million cells was determined to be the best option for first geometry (see Fig. 5).

### Make sure about accuracy

**Figure 6:**
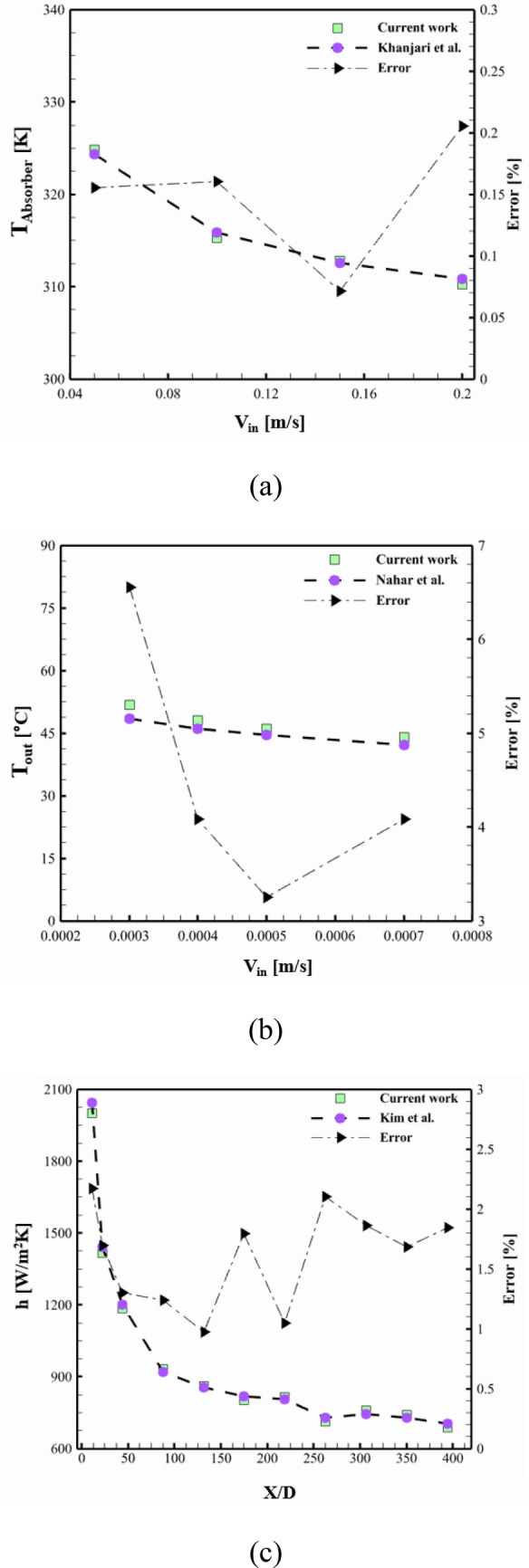
Comparison of the obtained outputs with the those of the work by (**a**) Khanjari et al.^40^ for Ag-water, (**b**) Nahar et al.^41^, and (**c**) Kim et al.^42^.

Validation is a critical step in numerical simulation as it ensures accurate representation of the system being analyzed. Numerical models are simplifications of complex physical systems, with inherent assumptions and limitations. Therefore, validation is essential to ensure that the simulation accurately captures the essential physical treatment of unit. The code was verified employing data from Khanjari et al.^40^, who scrutinized the influence of nanofluid and pure water on solar panel and they utilized a copper tube. By comparing the absorber temperatures, the outputs showed good agreement (see Fig. 6a). To validate the simulation further, the empirical study of Nahar et al.^41^ was used. In their study, the authors investigated a polycrystalline silicon PV panel's outdoor performance. The validation was based on *T*_*out*_, which demonstrated an error percentage of less than 6.6% (see Fig. 6b). The third validation step involved comparing the *h*_*x*_ value to the experiment performed by Kim et al.^42^, who used a tube with a 2 m length and a 4.57 mm diameter. The comparison revealed an error percentage of less than 3% for the entire dataset (see Fig. 6c). These three validation steps confirm that the chosen approach is reasonably accurate for modeling the current work.

### Selecting the best design of cooling duct

**Figure 7:**
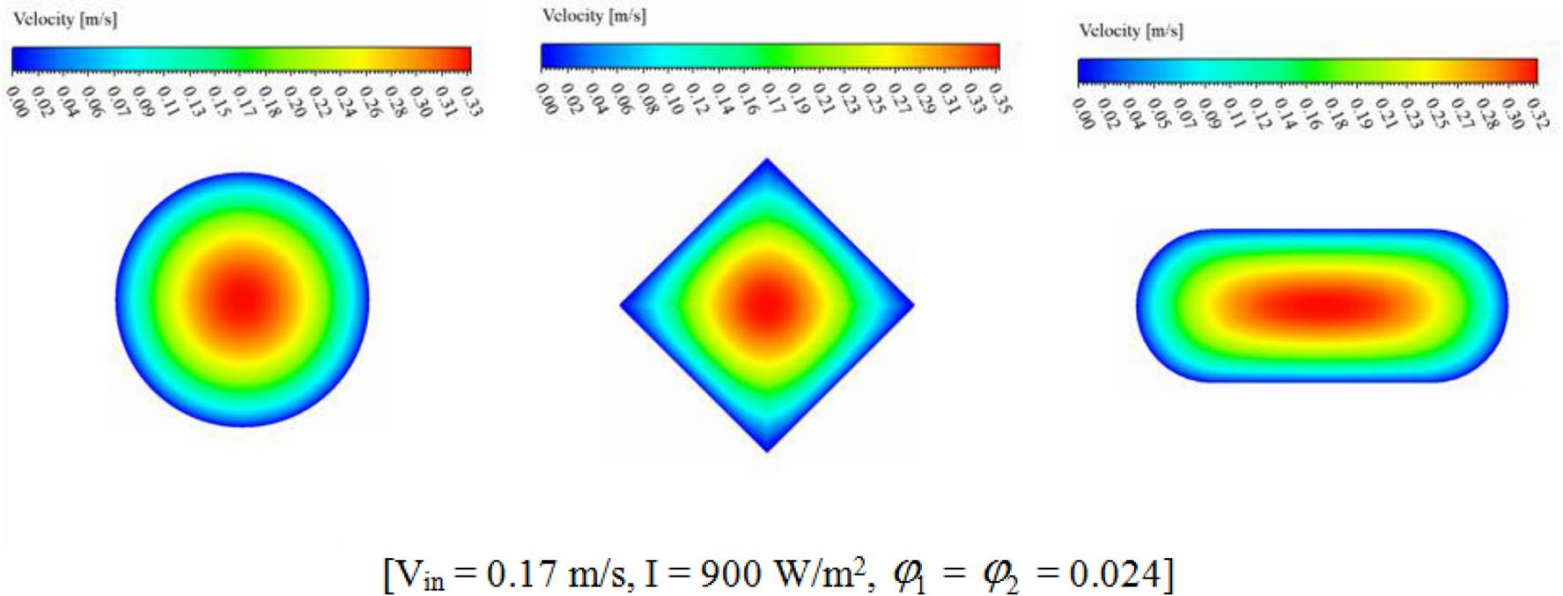
The contour of velocity at Z = 0.992 m.

**Figure 8:**
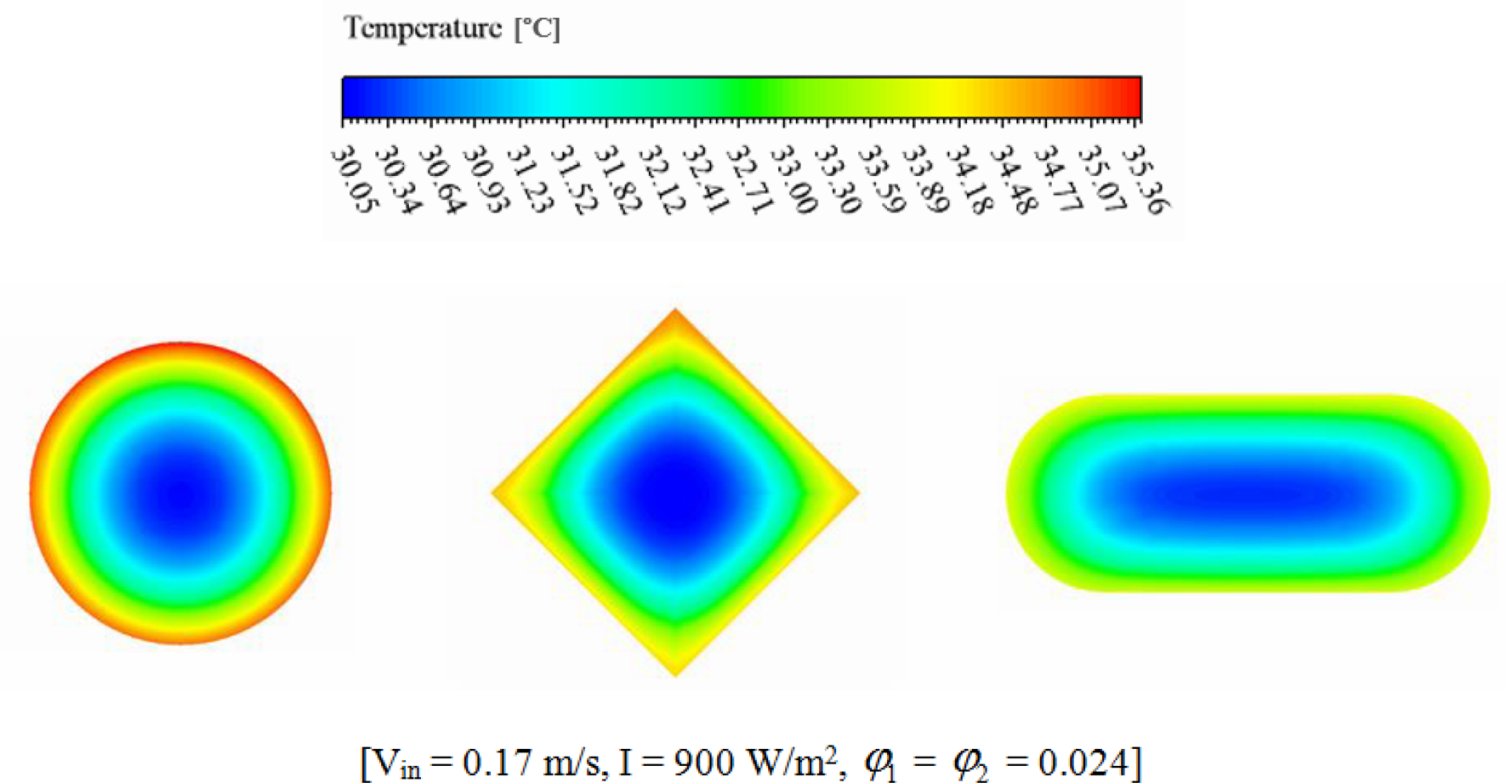
The contour of temperature at Z = 0.992 m.

**Figure 9:**
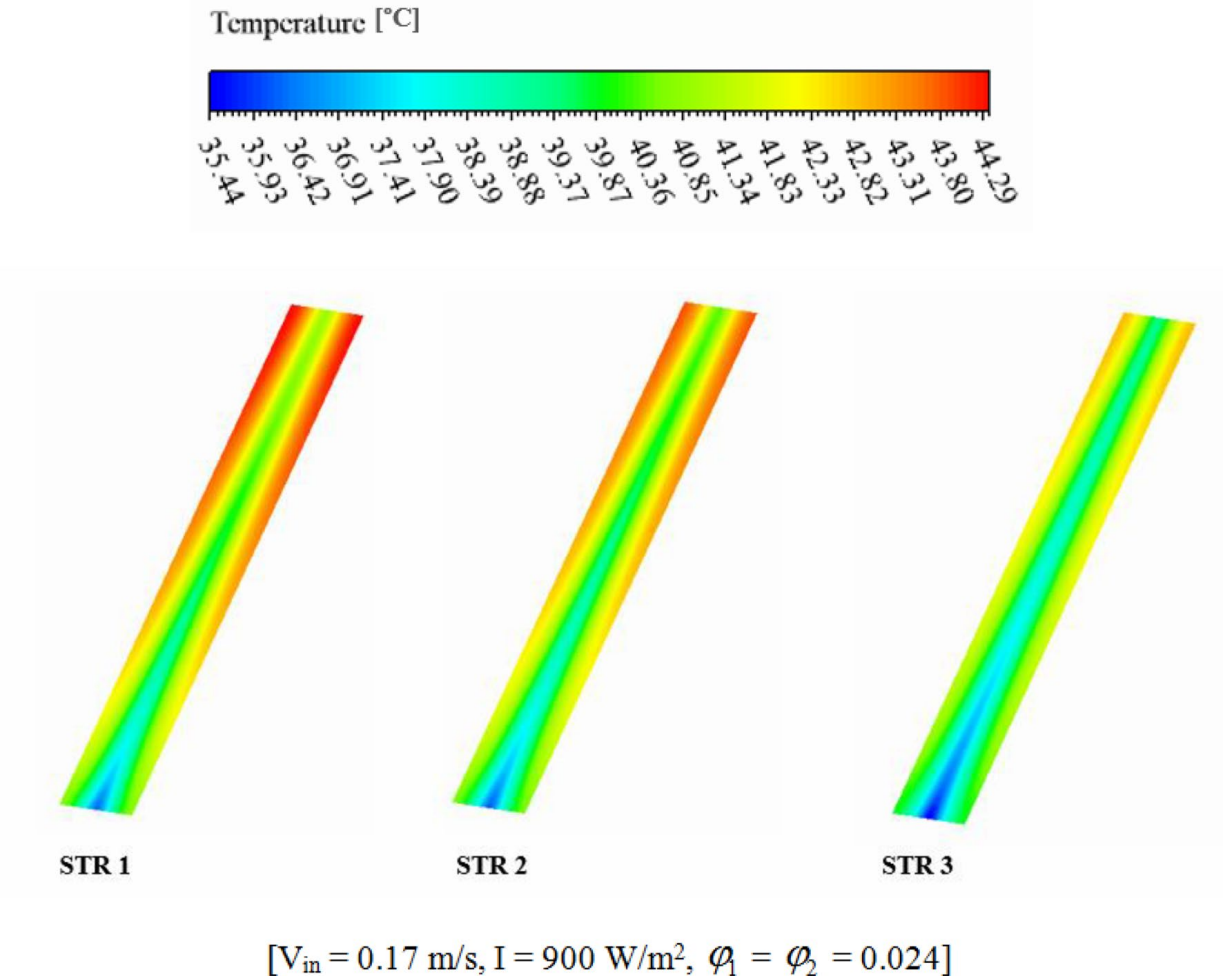
The contour of the PV temperature in three structures.

The efficiency of a PV unit attached to a tube with nanofluid can be influenced by the design of the tube. The geometry of the cross-section can impact the flow rate and distribution, which affects the system's efficiency. If the hydraulic diameter is fixed, changing the cross-sectional shape can alter the flow regime and enhance thermal performance. The employ of hybrid nanofluid can further enhance the system's heat transfer performance by increasing *k*_*nf*_. Thus, optimizing the tube's cross-sectional shape attached to a PV system with nanofluid can result in significant performance improvements, improving the system's efficiency and reliability. The dimensions of three scrutinized geometries for cross section cooling duct have been mentioned in Fig. 2. To reach the equal Reynolds number, the hydraulic diameters (*D*_*H*_) of all geometries are 0.0077 m. The range of Re for inlet velocity of 0.065 to 0.17 m/s are 498.1 to 1302.74 which means that laminar flow assumption is reasonable approximation. The circular cross-section results in uniform flow distribution, while two other cross-sections produce non-uniform flow, which can improve heat transfer rates. The cross section at Z = 0.992 m, has been presented for various geometry to show the velocity and temperature of hybrid nanofluid (see Figs. 7 and 8). The velocity of SRT 2 is greater than other geometries while maximum temperature of STR3 is the lowest. The distribution of temperature over the silicon layer has an important role on the life of the panel and related contours for various geometries have been shown in Fig. 9. According to values of maximum cell temperature, the minimum value can be obtained if STR3 has been selected. For third structure, the uniformity of contours is improved about 8.9% and 3.92% in comparison to STR1 and STR2, respectively.

**Figure 10:**
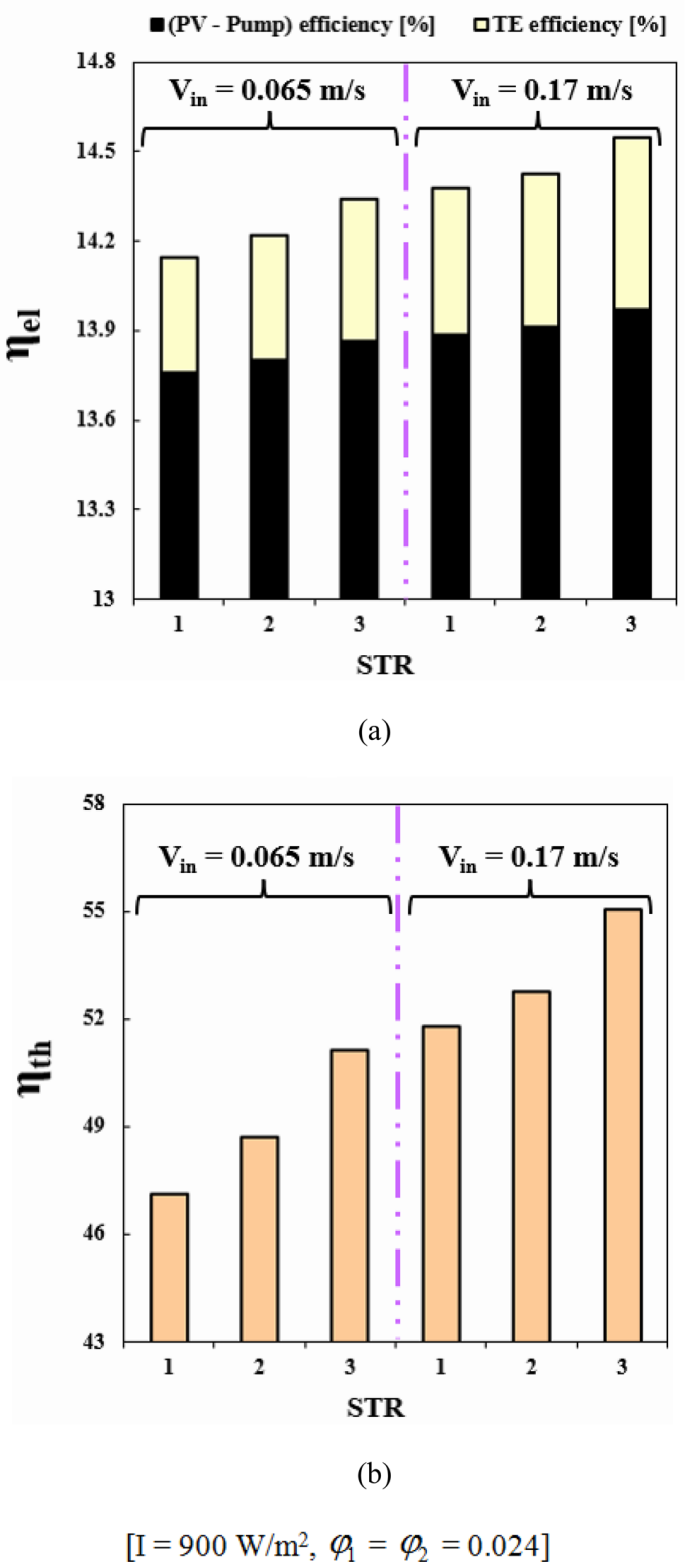
(**a**) *η*_*el*_ and (**b**) *η*_*th*_ in desired structures for the HTT.

Selecting the best geometry of the duct can lead to greater efficiency. This is because the duct's geometry affects the fluid flow, which in turn affects the efficiency. By selecting the best geometry, the fluid can flow more smoothly, reducing the cell temperature. This results in improved electrical and thermal performance, allowing for greater energy output from the PVT system. Among the scrutinized geometries, the best performance belongs to third structure (see Fig. 10). When V_in_ = 0.065 m/s, with switching from STR1 to STR3, *η*_*el*_ and *η*_*th*_ enhance about 1.39% and 4.83%, respectively. Also, for the same Re, changing structure form 2 to 3, *η*_*el*_ and *η*_*th*_ enhance about 0.86% and 5.03%, respectively. The summation of these two functions which can be named as overall efficiency, improving about 6.83% and 4.08% with replacing STR3 instead of STR1 and STR2, respectively. The increment of overall efficiency with changing from STR1 to STR3 decreases about 31.36% if inlet velocity enhances up to 0.17 m/s.

### Impact of inlet velocity for the best geometry of duct

**Figure 11:**
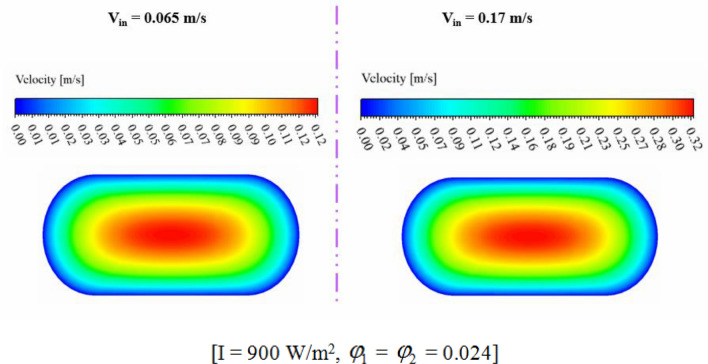
The contour of the fluid velocity at Z = 0.992 m.

**Figure 12:**
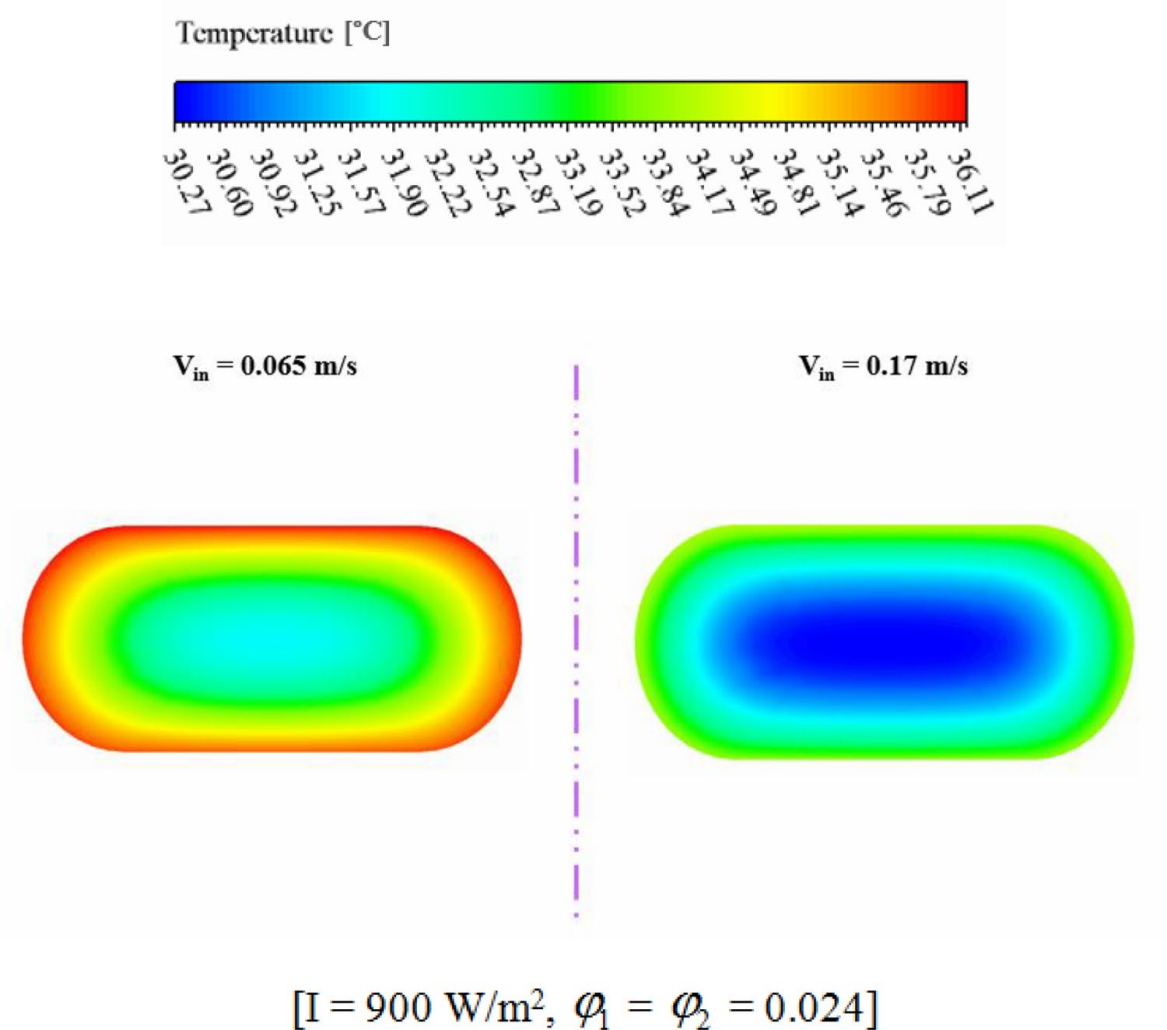
The efficacy of V_in_ on fluid temperature at Z = 0.992 m.

**Figure 13:**
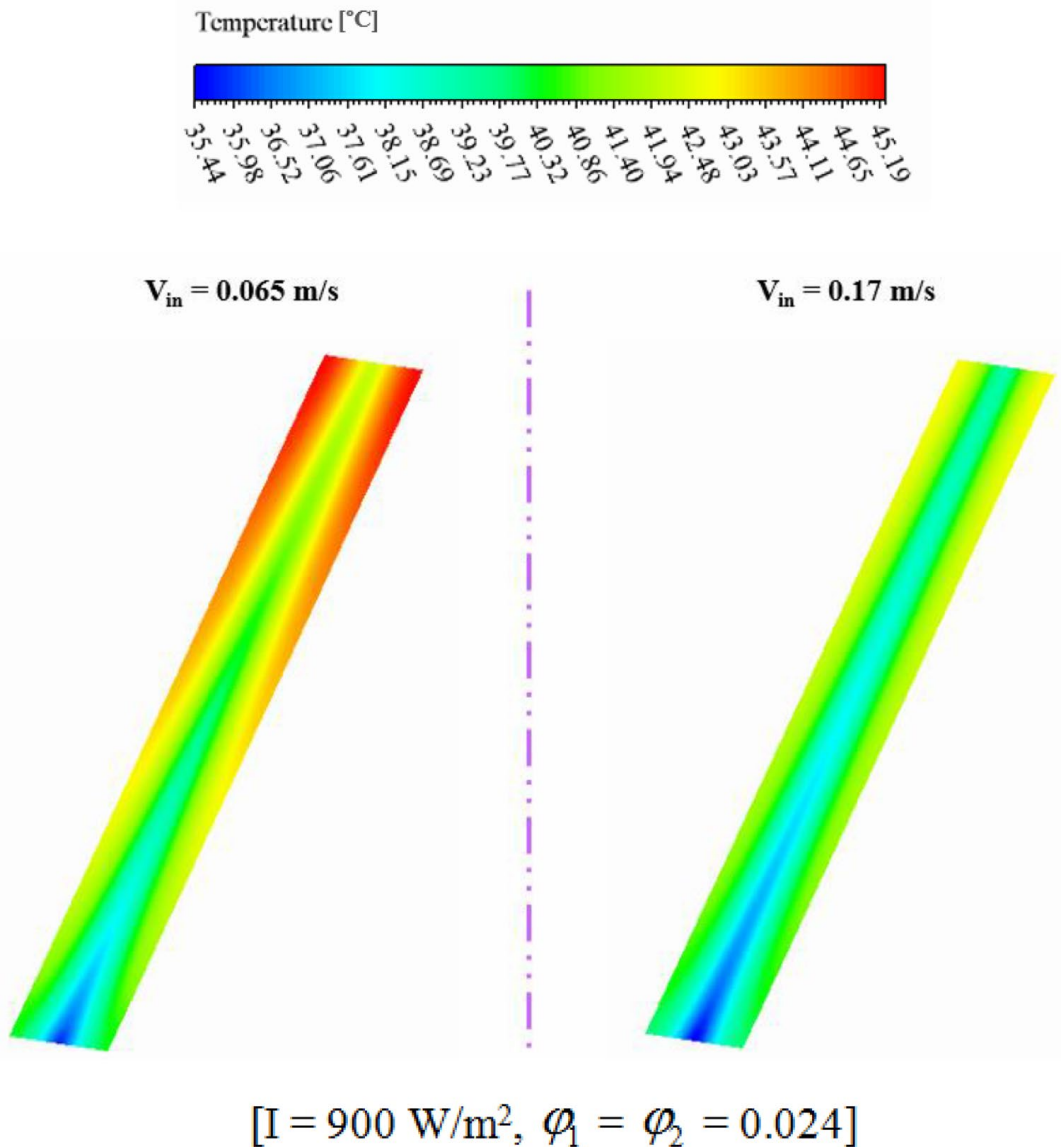
The efficacy of V_in_ on T_PV_.

**Figure 14:**
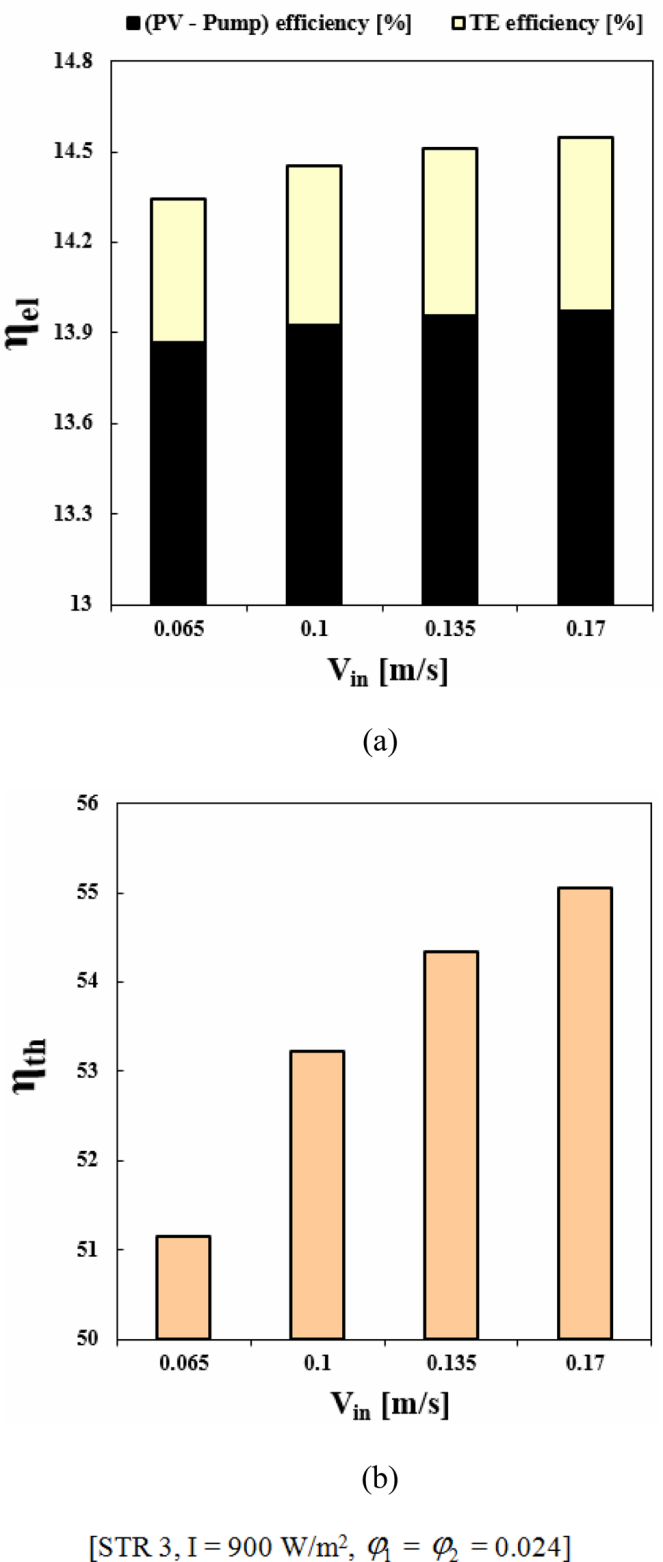
(**a**) *η*_*el*_ and (**b**) *η*_*th*_ at various fluid inlet velocities.

The impact of nanofluid inlet velocity on PVT system performance can be explained by physical mechanisms. An increase in velocity results in a greater convective flow coefficient, which lowers the panel temperature and raises useful heat. This leads to higher thermal and electrical efficiency. Higher velocities also improve hybrid nanofluid mixing and distribution within the duct, which further improves efficiency. However, excessive velocity can increase pressure drop and pumping power, leading to reduced performance and this effect has been involved in calculating *η*_*el*_. The hydrothermal behavior of hybrid nanofluid has been illustrated in Figs. 11 and 12. Maximum velocity of hybrid nanofluid at Z = 0.992 m increases about 2.66 times greater value while temperature of hybrid nanofluid decreases. As demonstrated in Fig. 13, the silicon layer temperature declines with growth of V_in_ and uniformity enhances about 21.1%. To show the influence of V_in_ on performance, Fig. 14 was demonstrated. With change of V_in_ from 0.065 to 0.1 and 0.135 m/s, the overall efficiency enhances about 3.34% and 5.14%, respectively. As velocity enhances from 0.065 up to maximum magnitude (0.17 m/s), *η*_*el*_ and *η*_*th*_ augment about 1.43% and 7.65%, respectively. The maximum values of *η*_*el*_ and *η*_*th*_ are 14.54% and 55.06% when *φ*_1_ = *φ*_2_ = 0.024 and V_in_ = 0.17 m/s.

### Influence of concentration of component of hybrid nanofluid

**Figure 15:**
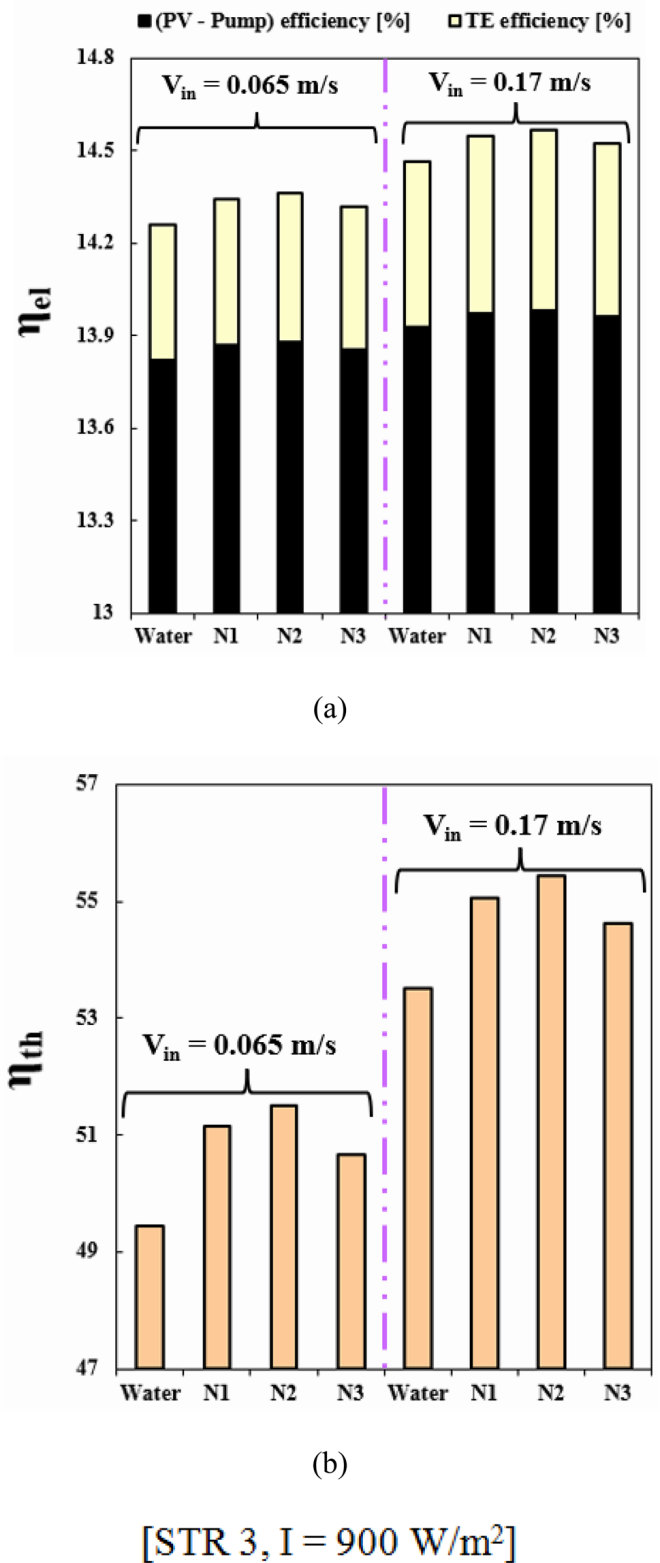
(**a**) *η*_*el*_ and (**b**) *η*_*th*_ for water and various volume concentration of the Fe_3_O_4_-SWCNT/water hybrid nanofluid.

The performance of unit is influenced by the fraction ratio of the two components of hybrid nanoparticles, Fe_3_O_4_ and MWCNT, within the water. The fraction ratio can change the features of the hybrid nanofluid, which in turn affects the efficiency of the PVT-TEG unit. So, it is crucial to explore and find the best fraction ratio of hybrid nanoparticles in the nanofluid to achieve the best performance of the PVT unit. As illustrated in Table 1, three conditions [N1 (*φ*_1_ = *φ*_2_ = 0.024), N2 (*φ*_1_ = 0.012, *φ*_2_ = 0.036), N3 (*φ*_1_ = 0.036, *φ*_2_ = 0.012)] have been tested and total fraction for all cases are 0.048 to satisfy the limitation of single phase approach. These three cases have been compared with case of pure water and outputs were demonstrated in Fig. 15. When V_in_ = 0.17, with change of ratio of fraction from N3 to N2, the amounts of *η*_*el*_ and *η*_*th*_ grow about 70.57% and 73.87%, respectively. When V_in_ = 0.065, adding hybrid nanoparticles with fractions of N1, N2 and N3 into water makes overall efficiency enhance about 2.78%, 3.39% and 2.01%, respectively. Among various cases, N2 has the greatest values of *η*_*el*_ and *η*_*th*_ for the condition of V_in_ = 0.17, these values are 14.56% and 55.42%, respectively. With growth of V_in_ for N2, the overall performance augments about 6.26%. The improvement of overall efficiency with adding hybrid nanoparticles (N2) in existence of V_in_ = 0.065 is about 13.88% greater than that of V_in_ = 0.17 m/s.

**Table 1:** The volume concentration of each nanofluid.

Nanofluid	*φ*_1_ (Fe_3_O_4_)	*φ*_1_ (SWCNT)
N1	0.024	0.024
N2	0.012	0.036
N3	0.036	0.012

### The efficacy of (“I”) on behavior of PVT-TEG

**Figure 16:**
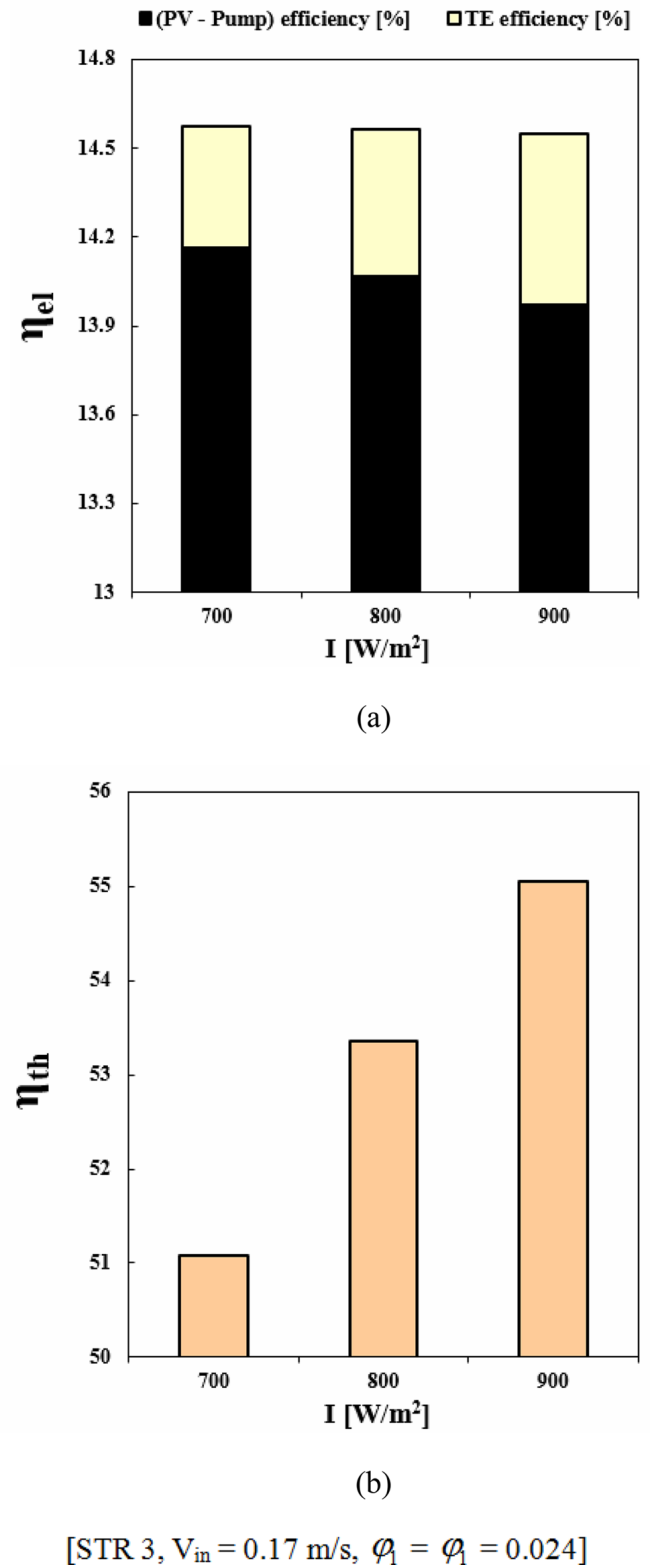
(**a**) *η*_*el*_ and (**b**) *η*_*th*_ at three levels of the solar irradiance.

The amount of solar irradiation (“I”) can increase and enhance the overall perfomance of a PVT-TEG system. With employing greater values of “I”, the output power is enhanced but the significance of better cooling techniques become more sensible. To show the influence of “I” on performance of the system, three levels of this factor have been applied and associated outputs were illustrated in Fig. 16. With an increase of “I” from 700 to 900 W/m^2^, *η*_*el*_ reduces about 0.18% while *η*_*th*_ augment around 7.79%. The value of *η*_*th*_ augments from 51.07% to 55.06% when solar irradiation increases from 700 to 900 W/m^2^. Although, the TEG performance enhances about 40.17% with growth of “I”, the overall electrical efficiency decreases because of reduced PV performance owing to the increment of silicon layer temperature.

### The improvement in electrical performance compared to an uncooled system

**Figure 17:**
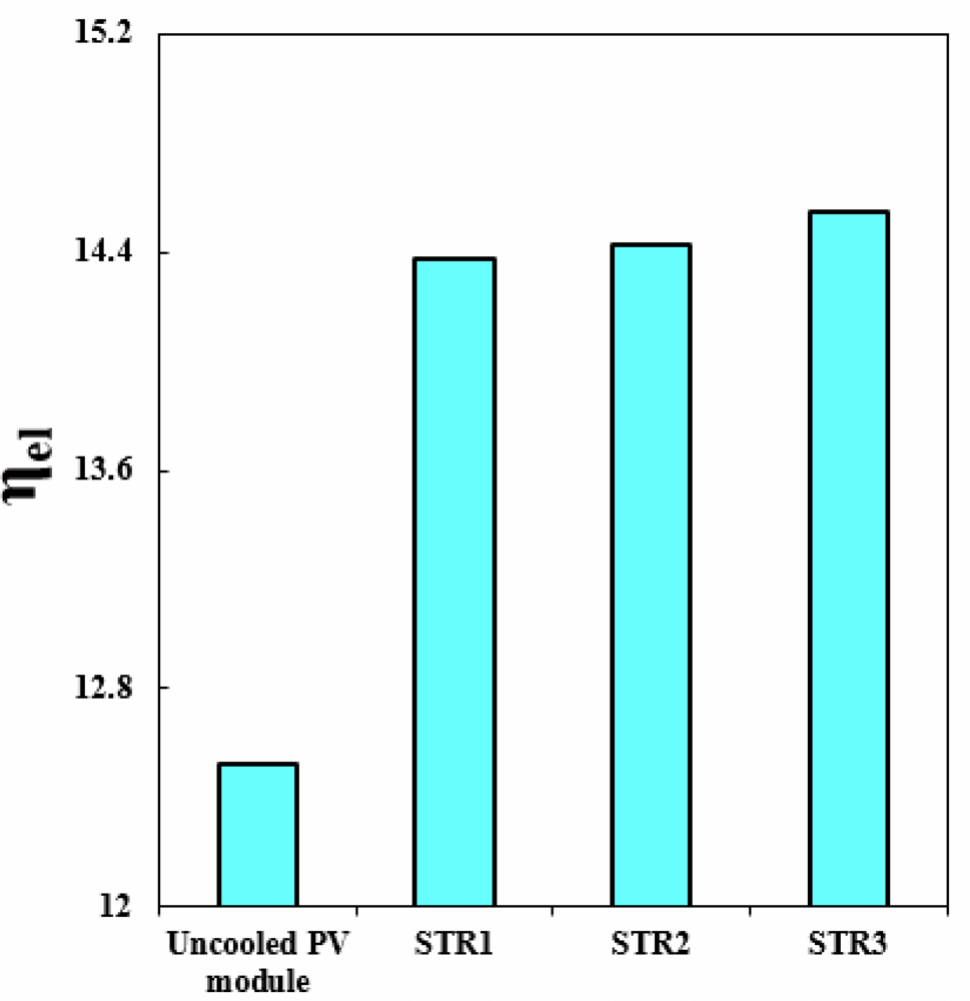
The improvement of *η*_*el*_ compared to an uncooled PV module.

In order to compare the improvement of *η*_*el*_, the outputs for various structures of the cross sections have been compared with the uncooled module. Figure 17 depicts the related outputs to show the promising influence of utilizing cooling systems. The concentration of hybrid nanofluid for these outputs is *φ*_1_ = *φ*_2_ = 0.024. The enhancement of *η*_*el*_, for STR1, STR2 and STR3 are 14.82%, 15.22% and 16.2%, respectively. This result indicates that the third geometry has the greatest promising effect of *η*_*el*_.

### The percentage of improvement in efficiency compared to previous works

**Figure 18:**
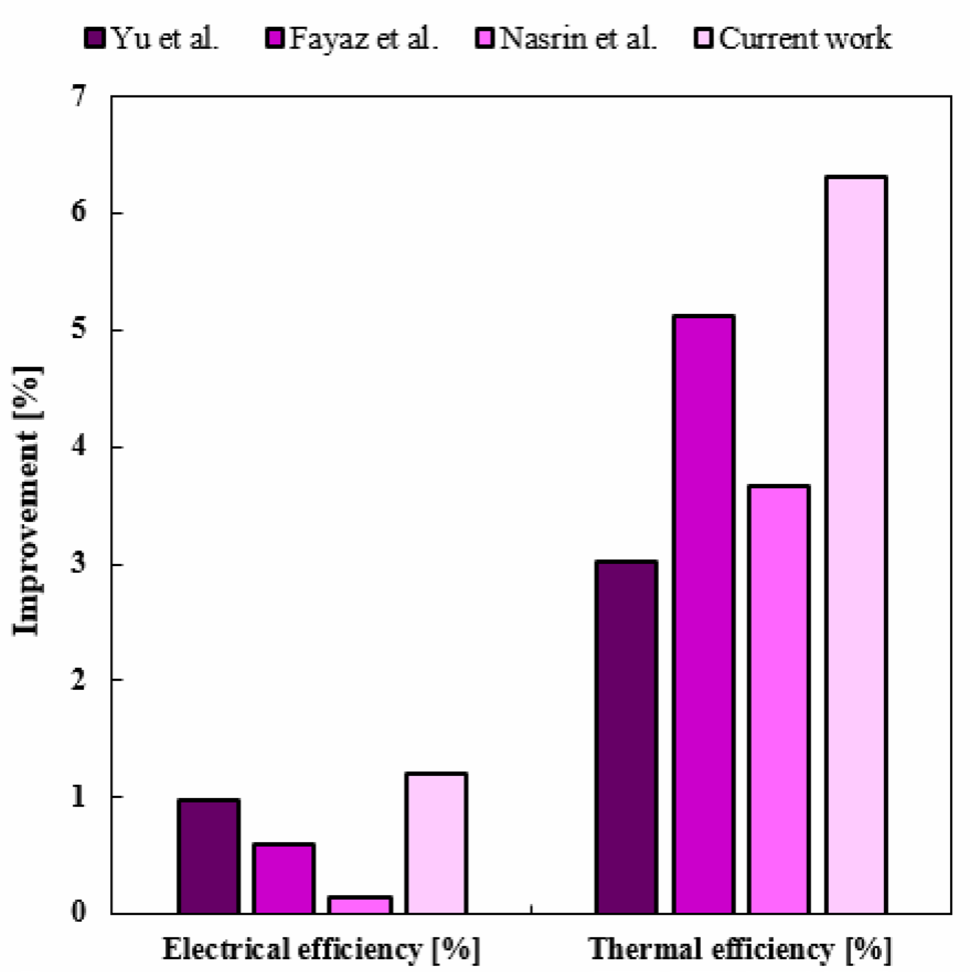
The improvement of *η*_*el*_ and *η*_*th*_ compared to the recent publications.

The previous studies reported the rate of improvement for both functions of *η*_*el*_ and *η*_*th*_ in comparison with their basic case. These percentages of improvement can be compared with present rate of improvement. So, Fig. 18 has been prepared to show the comparison of the enhancement of performance with previous works (Yu et al.^43^, Fayaz et al.^44^, Nasrin et al.^18^). The increments of *η*_*el*_ for works of^43,44^ and^18^ are 0.97%, 0.6% and 0.14%. For the current work, *η*_*el*_ enhances about 1.2% in comparison with base case (STR1). Moreover, the augmentation of *η*_*th*_ for works of^43,44^ and^18^ are 3.02%, 5.13% and 3.67%, respectively. The increment of *η*_*th*_ for current work is about 6.31% which is greater than other previously mentioned studies.

## Conclusions

To boost the performance of PVT units, cooling tubes with various configurations have been scrutinized in the current article. To enhance the cooling rate, the base fluid has been mixed with hybrid nanoparticles and influence of various fraction ratios has been compared. To use the waste heat, TEG layer has been combined with other layers of PV leading to increment of electrical performance. The negative impact of pumping power has been involved in measuring the overall electrical performance. Three different geometries (STR1 (circular), STR2 (rhombus), STR3 (elliptic)) have been tested to find the best design. All geometries have the same inlet Reynolds number and laminar flow has been considered through the tube. With improvement of cooling rate and temperature uniformity with loading nanoparticles and selecting the best design, the thermal stress over the panel decreases leading to higher lifespan. The impacts of inlet velocity (V_in_ = 0.065 to 0.17) and different fractions of Fe_3_O_4_ and MWCNT have been examined. The optical features have been involved in modeling by incorporating the heat generation terms for layers. Due to the negligible value of heat source in layers at the beneath of second EVA, the pure conduction mode without heat source has been considered for them. The properties of new working fluid have been estimated according to single phase approach. To increase stability in numerical simulations, structured grid has been applied for all geometries and grid independency technique has been presented. The best number of cell for STR1 is 3.3 million. According to simplifications and assumptions of simulation, it is essential to present validation step. Three steps have been presented in result section as a validation procedure. Not only previous numerical publications but also experimental data have been evaluated and good accuracy has been achieved. The role of geometry of cooling duct has been examined and associated outputs in view of contours and bar charts have been presented. Due to changing the style of flow for various geometries, the cooling rate has been changed and performance of system can be improved with selecting the best design. The uniformity of silicon layer temperature improves around 8.9% and 3.92% with replacing STR3 instead of STR1 and STR2. Given V_in_ = 0.065, with changing structure from first to third, *η*_*el*_ and *η*_*th*_ enhances about 1.39% and 4.83%, respectively. Overall efficiency enhances about 6.83% and 4.08% with replacing STR3 instead of STR1 and STR2, respectively. The velocity of fluid can enhance the performance of system because the silicon layer temperature can decrease with growth of V_in_. The uniformity of silicon layer temperature can improve about 21.1%. As V_in_ grows from 0.065 to 0.17 m/s, *η*_*el*_ and *η*_*th*_ enhance about 1.43% and 7.65%, respectively. The greatest amounts of *η*_*el*_ and *η*_*th*_ are 14.54% and 55.06% when *φ*_1_ = *φ*_2_ = 0.024 and V_in_ = 0.17 m/s. To show the influence of fraction ratio of components of hybrid nanoparticles, three cases have been compared with case of water and outputs showed that the case N2 (*φ*_1_ = 0.012, *φ*_2_ = 0.036) has the best performance. When V_in_ = 0.17, the values of *η*_*el*_ and *η*_*th*_ for N2, reached 14.56% and 55.42%, respectively. Given V_in_ = 0.065, dispersing hybrid nanoparticles with fractions of N1, N2 and N3 into water causes overall efficiency to increase around 2.78%, 3.39% and 2.01%, respectively. The increments of *η*_*el*_ and *η*_*th*_ in comparison of base case (STR1) are about 1.2% and 6.31%, respectively. These percentages are greater than previous scrutinized articles. The comparison of value of *η*_*el*_ with an uncooled system showed that the performance enhances about 14.82%, 15.22% and 16.2%, for STR1, STR2 and STR3, respectively. As solar irradiance enhances, the value of *η*_*el*_ reduces about 0.18% while *η*_*th*_ augments around 7.79%.

## Data Availability

All data generated or analysed during this study are included in this published article.
