# Author Correction: Hybrid nanofluid flow within cooling tube of photovoltaic-thermoelectric solar unit

**DOI:** 10.1038/s41598-023-38125-6

**Published:** 2023-07-10

**Authors:** Z. Khalili, M. Sheikholeslami, Ladan Momayez

**Affiliations:** 1 Department of Mechanical Engineering, Babol Noshirvani University of Technology, Babol, Islamic Republic of Iran; 2 Renewable Energy Systems and Nanofluid Applications in Heat Transfer Laboratory, Babol Noshirvani University of Technology, Babol, Islamic Republic of Iran; 3 Department of Engineering and Computer Science, University of Pittsburgh at Johnstown, Johnstown, PA USA

Correction to: *Scientific Reports* 10.1038/s41598-023-35428-6, published online 21 May 2023

The original version of this Article contained an error in Affiliation 3.

“Department of Mechanical and Aerospace Engineering, University of Pittsburgh, Pittsburgh, USA.”

now reads:

“Department of Engineering and Computer Science, University of Pittsburgh at Johnstown, Johnstown, PA, USA.”

The original Article has been corrected.

